# Clinical and Patient-Related Outcome After Stabilization of Dorsal Pelvic Ring Fractures: A Retrospective Study Comparing Transiliac Fixator (TIFI) and Spinopelvic Fixation (SPF)

**DOI:** 10.3389/fsurg.2021.745051

**Published:** 2021-11-29

**Authors:** Ricarda Johanna Seemann, Erik Hempel, Gabriele Rußow, Serafeim Tsitsilonis, Ulrich Stöckle, Sven Märdian

**Affiliations:** ^1^Center for Muskuloskeletal Surgery, Charité - University Medicine Berlin, Corporate Member of Freie Universität Berlin, Humboldt-Universität zu Berlin, and Berlin Institute of Health, Berlin, Germany; ^2^Julius Wolff Institute for Biomechanics and Musculoskeletal Regeneration, Berlin, Germany

**Keywords:** dorsal pelvic ring fractures, transiliac fixator, spinopelvic fixation, outcome, PROMS

## Abstract

**Purpose:** Aim of this retrospective cohort study was the comparison of the transiliac fixator (TIFI) and spinopelvic fixation (SPF) for fixation of dorsal pelvic ring fractures in terms of clinical outcome, complications, and quality of life.

**Methods:** Thirty-eight patients (23 men, 15 women; mean age 47 ± 19 years) with dorsal pelvic ring fractures (type-C-injuries after AO/OTA) that have been stabilized by either TIFI (group TIFI, *n* = 22) or SPF (group SPF, *n* = 16) between May 2015 and December 2018 were retrospectively reviewed. Outcome measurements included demographic data, perioperative parameters, and complications and were obtained from the medical information system. Quality of life was assessed using the German version of the short form 36 (SF-36) and short muskuloskeletal function assessment (SMFA-D). Clinical results were assessed using Merle d'Aubigné-Score, Iowa Pelvic Score, and Majeed Pelvic Score.

**Results:** Both groups show relatively good post-operative results, which has previously been reported. Quality of life was comparable in both groups. Group TIFI was slightly superior regarding complication rates, cutting/suture time, and fluoroscopy time. Group SPF seemed to be superior regarding pain and pelvic scores.

**Conclusion:** None of the methods could demonstrate significant superiority over the other. Management of pelvic injuries remains a highly individual challenge adapted to the individual patients' condition. Nevertheless, if fractures allow for stabilization with TIFI, the use of this method should be taken into consideration as a less invasive and more tissue-conserving approach.

## Introduction

The incidence of pelvic ring fractures—especially in elderly patients—has continued to rise during the last decades (1). While the overall trauma mortality steadily decreased over the last decades, pelvic ring injuries—particularly complex forms following high energy trauma—are still associated with increased mortality rates of up to 18% (2, 3). To date, the outcome of these serious injuries is often unsatisfactory (4). Fractures of the pelvis are intimately connected with a significant drop in this patient group's quality of life (QoL) (5). Besides, a large cohort of patients suffers from these injuries in their most productive age, resulting in an enormous socioeconomic burden. Due to the ongoing demographic change, the incidence of fractures of the pelvic ring is expected to rise, which will be even more pronounced in elderly patients (1, 6), who remain active in their daily activities despite their age. Thus, there is a significant need to develop treatment algorithms and osteosynthetic options further. Different fixation options have been published in the literature (7–13) and have partly undergone intense biomechanical investigations (14–18). However, it is still a considerable debate among pelvic surgeons, which might be the best approach to stabilize the posterior pelvic ring. While the management approach for clinically unstable patients in the Emergency Department (ED) is mainly standardized today (e.g., ATLS^®^ algorithm), there is no consensus on stabilization options for accompanying pelvic fractures. Percutaneous sacroiliac (SI) screws are widely used to fix posterior pelvic ring fractures (19), although their purchase in osteoporotic bone is limited. Alternative methods, especially in patients with higher degrees of instabilities of the pelvic ring, are the spinopelvic fixation (SPF) and the transiliac fixator (TIFI) (7–10), both of which may be applied in a minimally invasive or open manner (20–24).

Both described techniques showed similar or higher biomechanical stability in cadaveric studies than one or two SI-screws (14–17, 25). According to the AO / Orthopedic Trauma Association (OTA) classification (26), both type B and type C fractures might be stabilized with either method. Although these surgical methods are clinically well established, outcome data remain rare. Kerschbaum et al. found a significantly reduced patient-reported outcome following TIFI or SPF compared to a healthy reference population (27) but did not provide clinical follow-up. Advantages of the TIFI include less time to apply, lower invasiveness and the exclusion of the caudal lumbar segments in the fixation construct. To our best knowledge, studies comparing the clinical outcome of SPF vs. TIFI do not exist. Therefore, this study aimed to compare these two fixation methods regarding the functional outcome, mechanical complications, and life quality.

## Materials and Methods

**Figure 1:**
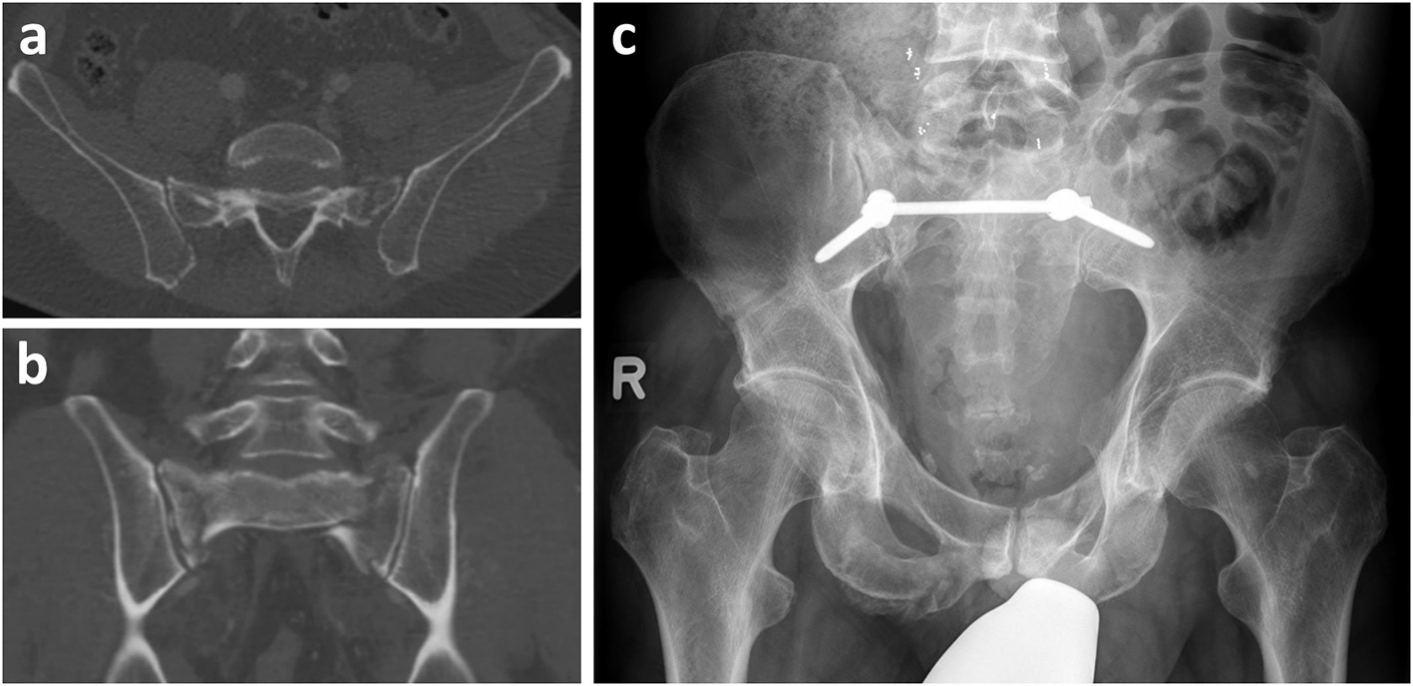
TIFI. Male patient (53 years old) after fall from greater height. Pelvic injury was classified (**a:** CT scan coronal, **b:** transversal) as AO C2.1b2c3. After initial stabilization with external fixator, definitie surgery was performed with transiliac fixation **(c)**.

**Figure 2:**
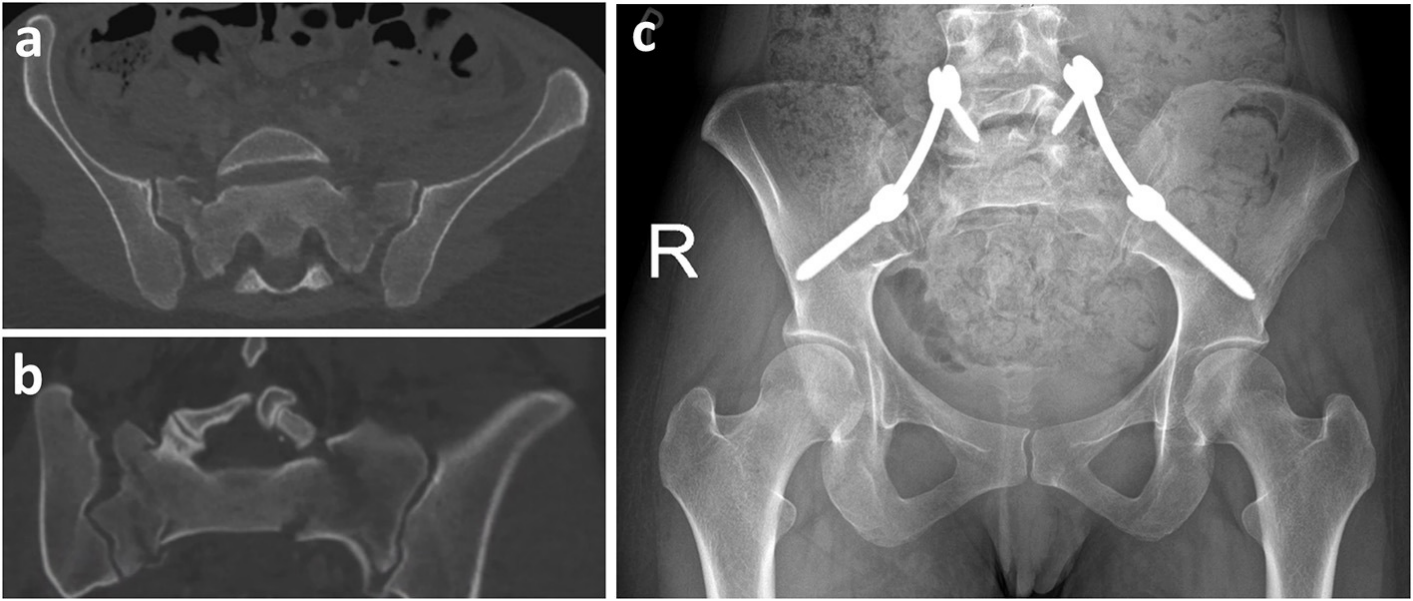
SPF. Female patient (29 years old) with spinopelvic dissociation and bilateral sacral fracture after road accident. The injury was classified (**a:** CT scan coronal, **b:** transversal) as AO C3.3. Surgery was performed with spinopelvic fixation (SPF) (L5 to Os ilium) **(c)**.

**Figure 3:**
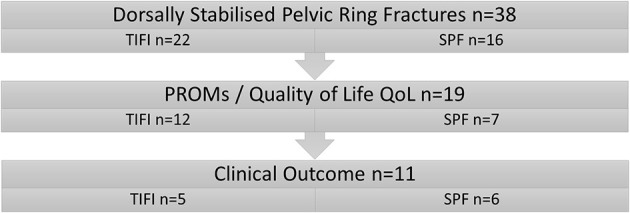
Patient cohort. Patients were separated into two groups according to the surgical procedure.

All patients who suffered a pelvic fracture involving the posterior pelvic (classified as type C according to AO/OTA) ring between May 2015 and December 2018 that were operatively stabilized by the senior pelvic surgeon using either TIFI (group TIFI, Figure 1) or SPF (group SPF, Figure 2) were included in this retrospective study. This resulted in thirty-eight patients (23 men, 15 women) with a mean age of 47 ± 19 years (Figure 3).

### Surgical Technique

Patients were put in a prone position on the radiolucent table. AP and lateral fluoroscopy views were obtained to control for the quality of reduction. The two surgical techniques used were the transiliac internal fixator (iliac screw–rod–iliac screw, Figure 1) and spinopelvic fixation SPF (iliac screw–connecting rod–L5 screw, Figure 2). The decision to select SPF vs. TIFI was mainly based on the intraoperative justification of the instability of the fracture. In cases of iliosacral dislocation, a TIFI was consistently applied. Correct screw placement was confirmed with an intraoperative 3D scan.

PROMs (patient-reported outcome measures, e.g., results of quality of life (QoL) questionnaires) could be obtained in 19 patients (eight refused functional follow-up, but gave consent for a phone interview) with a mean follow up 26.1 ± 14.3 months. Additional functional outcome data could be obtained in 11 patients (mean follow up 17.2 ± 7.9 months). Of the remaining 19 patients, 11 patients moved to an unknown address. Seven did not give consent for the additional follow-up *via* telephone and clinical examination. One patient had died for reasons not connected to the study.

Demographic data, intra- and post-operative details, including the timing and duration of surgery, pre- and post-operative CT scans, as well as complications, were obtained from the hospital's medical information system. Complications were graded according to Dindo et al. (28) and divided into major (Grade III and above, e.g., mechanical failure, infections, revision surgery) and minor (Grade I and II, e.g., thromboembolic events and pneumonia). Fractures were classified according to AO/OTA. QoL was assessed using the German version of the short form 36 (SF-36) (29). Raw data transformation and summary score calculations were performed as described by Bullinger et al. (30, 31). Normative data from Germany (7525 persons) were used for comparison (32). Furthermore, the SMFA-D was used to rate the functional aspect of QoL (33, 34). Functional results were assessed using Merle d'Aubigné-Score (35), Iowa Pelvic Score (36) and Majeed Pelvic Score (37).

The study was adherent to the local institutional review board and the ethics commissioner's vote (No. EA2/036/16). All data were recorded and analyzed using IBM-SPSS Statistics Release 25.0 (IBM, Armonk, New York). The assumption of normality and homogeneity of variance was tested using the Kolmogorov-Smirnov test. Statistical analysis involved the *t*-test for numerical matched/unmatched samples. The chi-squared test was used for cross table evaluation. Differences were considered significant at a *p*-value < 0.05.

## Results

### Demographics and Surgical Data

Twenty-two patients (*m*:*w* 15:7; mean age: 50 ± 20 years) were treated with TIFI and 16 patients (*m*:*w* 8:8; mean age: 43 ± 16 years) with SPF (see Table 1). Comparability of the groups was assumed as there were no significant differences between the two groups concerning gender (*p* = 0.234), age (*p* = 0.271), and cause of injury (*p* = 0.502). Most injuries occurred due to falls from great heights (58%), followed by traffic incidents (29%). Five patients were treated for low energy traumata. The surgical details recorded are given in Table 2.

**Table 1:** Demographic data.

**Demographic data**	**TIFI *n* = 22**	**SPF *n* = 16**
**Gender distribution**
*Male n* = *23*	15	8
*Female n* = *16*	7	8
**Age at the time of surgery (years)**	50 ± 20	43 ± 16
**AO/OTA fracture classification**
*Type C1 n* = *14*	13/38	1/38
*Type C2 n* = *6*	4/38	2/38
*Type C3 n* = *18*	5/38	13/38

**Table 2:** Summary of recorded surgical details and complications.

**All patients *n* = 38**	**TIFI *n* = 22**	**SPF *n* = 16**	***p*-value**
Duration of isolated dorsal surgery (min)	107 ± 0 *(n* = *2)*	146.8 ± 52 *(n* = *5)*	0.542
Duration ventrodorsal combined surgery	221 ± 87 (*n* = 15)	285 ± 153 (*n* = 15)	0.171
Hospitalization (days)	33 ± 18	31 ± 16	0.685
*Major complications*			
heamatoma/ wound healing disturbances (*n* = 3)	0 (0%)	3 (18.8%)	
*Minor complications*			
Pneumonia (*n* = 7)	3 (13.6%)	4 (25%)	0.066
Thromboembolic events	2 (9%)	2 (12.5%)	
(*n* = 4)		

*Surgical details were tested using the t-test for unmatched samples; complications were evaluated using the chi-squared test for cross table evaluation. Values are given as mean (range; SD) or absolute numbers with percentage*.

We found no significant differences regarding the functional outcome, quality of life assessment or complication rate. Nevertheless, the TIFI group had a tendency toward less OR time (221 ± 87 min. vs. 285 ± 40 min, *p* = 0.171). Overall complication rate was 22.7% for the TIFI group and 50% in the SPF group. However, all complications in the TIFI group were graded minor whereas 18.8% of the complications in the SPF groupd were graded major. Statistical analysis showed that the TIFI group had a tendency towars less complications (*p* = 0.066). Table 2 gives a detailed overview of the complications. Clinical follow up revealed a tendency toward lower pain levels and higher scores in the functional outcome assessment with regard to the SPF group. Table 3 shows the detailed results of the clinical evaluation.

**Table 3:** Pain and clinical scores (Merle, Iowa, Majeed) after TIFI or SPF.

**Clinically assessed patients**	**TIFI *n* = 5**	**SPF *n* = 6**	***p*-value**
***n* = 11**			
Pain at rest (VAS 0–10)	4.2 ± 2.4	1.3 ± 1.4	0.082
Merle d'Aubigné	7.6 ± 3.4	10.8 ± 0.8	0.082
IOWA pelvic score	67.4 ± 24.6	86.2 ± 16	0.247
Majeed pelvic score	54.2 ± 25.6	83.3 ± 13.2	0.082

*Clinical results were evaluated using the chi-squared test for cross table evaluation. Values are given as mean (range; SD)*.

There were no significant differences between group TIFI and group SPF in the PROMs/QoL assessment. For SF-36, the global scores PCS/MCS and the subgroups were comparable (see Table 4). In concordance, the SMFA-D did not show any significant differences for the various indexes.

**Table 4:** Quality of life (SF-36 and SMFA-D) after TIFI or SPF.

**QoL patients *n* = 19**	**Normative value**	**TIFI *n* = 12**	**SPF *n* = 7**	***p*-value**
**Short form (36)**	**German population**	
PCS	51.4 (51.1–51.7)	38.5 ± 14.6	39.5 ± 14.0	0.773
Physical functioning	86.6 (86.0–87.2)	57.1 ± 32.9	62.9 ± 28.6	0.711
Physical role functioning	82.1 (81.3–82.8)	39.6 ± 39.1	32.1 ± 47.2	0.432
Bodily pain	74.8 (74.1–75.6)	53.4 ± 41.7	65.9 ± 32.7	0.902
General health perceptions	69.3 (68.7–69.9)	50.6 ± 23.9	55.0 ± 26.1	0.592
MCS	49.3 (49.0–49.6)	41.8 ± 12.1	46.9 ± 13.9	0.384
Vitality	61.6 (61.0–62.1)	58.1 ± 25.0	61.1 ± 21.5	0.773
Social role functioning	86.1 (85.4–86.7)	59.7 ± 36.2	80.6 ± 32.8	0.142
Emotional role functioning	86.0 (85.3–86.6)	60.8 ± 37.8	44.3 ± 52.2	0.592
Mental health	72.9 (72.4–73.4)	55.9 ± 24.4	68.1 ± 24.5	0.261
**SMFA-D**	**American population**	
Daily activities	11.9 ± 19.2	32.9 ± 29.4	31.4 ± 32.9	0.711
Emotion	20.5 ± 18.4	32.4 ± 19.1	25.5 ± 23.5	0.592
Arm-hand	6.0 ± 12.3	12.5 ± 20.0	6.2 ± 10.0	0.592
Mobility	13.6 ± 18.3	36.6 ± 26.9	26.6 ± 25.7	0.384
Dysfunction index	12.7 ± 15.6	29.0 ± 21.6	23.0 ± 22.0	0.536
Bother index	13.8 ± 18.6	33.0 ± 23.8	24.7 ± 20.0	0.432

*SF-36 and SMFA-D were evaluated using the chi-squared test for cross table evaluation. Values are given as mean (range; SD) or absolute number with 95 confidence interval*.

## Discussion

To our best knowledge, this is the first study to directly compare the transiliac internal fixator vs. a spinopelvic fixation for the osteosynthetic reconstruction of posterior pelvic ring instabilities. Our data reveal that SPF tended to have better clinical results. However, TIFI showed a tendency toward lower complication rates and favorable perioperative parameters (e.g., surgery duration). The mean age of patients included in our study corresponds with existing literature (3, 27, 38, 39), emphasizing pelvic ring fractures as injuries occurring in mostly young patients of employable age. In the study of Kerschbaum et al., patients underwent assessment *via* mail or telephone. Hence, clinical scores were not collected (27). The reported follow-up rate of 57.1% is only slightly higher than our 50% when only looking at the 19 patients asked for PROMs/quality of life. Our subgroup of eleven clinically assessed patients was comparable to the overall cohort regarding age and gender distribution. With 58%, falls from great heights were the leading cause of injury in our study population, whereas other authors published traffic accidents as the main reason (10, 38). One of the reasons for this might be the geographic location of our center in Berlin. Falls from great heights (high number of construction areas nearby) and suicide jumps occur pronouncedly in urban areas. Our treatment algorithm intends for a 360° stabilization of the pelvic ring in one procedure (depending on the patients' physiologic state), especially regarding the type C fractures that were analyzed here.

Thus, the surgical details of this study are not directly comparable to the data published earlier. In cases of an anterior and posterior procedure, surgery and fluoroscopy time documented represent the sum of both procedures. The cases with isolated stabilization of the posterior pelvic ring (TIFI: *n* = 2, SPF: *n* = 5) showed lower surgery times for the TIFI group (107 ± 0 min vs. 146.8 ± 52 min; *p* = 0,542). In this context, it has to be mentioned that our surgical technique of the TIFI differs from other publications in terms of screw placement, limiting comparability to published data. In concordance to our results, SPF surgery times in literature vary between 137 and 345 min (20–22, 24). The radiation time of both techniques in our cohort was comparably high (TIFI: 8.2 min; SPF: 12.1 min; *p* = 0.098). In contrast to published data, we used an intraoperative 3D scan to confirm correct screw placement (9, 22). A recent study by Hoffmann et al. showed comparable radiation times in conventional screw placement procedures. However, they also showed that navigation decreased radiation significantly. At the same time, OR time was longer compared to our procedures which may be explained by the need for setting up the navigation system (40).

Our data revealed a lower complication rate for the TIFI. Even if these results did not differ significantly, the absolute numbers seem to justify the assumption that TIFI tendentially yields fewer complications. No patient in the TIFI group but three following SPF (18.8%) had to be re-operated due to wound healing disorders/hematoma. This compares well to current literature, where complication rates regarding wound healing disorders/infections for TIFI of 6% and up to 35% for SPF are published (10, 21–24, 41–44). This might be a result of the more extensive surgical trauma, which is necessary to apply an SPF as compared to the TIFI.

As for pneumonia in our collective, three of 22 patients developed pneumonia (13.6%) after TIFI, compared to four of 16 patients (25%) after SPF. For TIFI, this result is consistent with the literature (44). However, the SPF group's rate of pneumonia was comparably high to reported rates (up to 9%) (45, 46). This might be due to a slower post-operative mobilization of the patients based on the more considerable surgical trauma. Our data regarding thromboembolic events for both groups TIFI and SPF is consistent with published literature (44–47).

There is evidence that post-traumatic pain affects the quality of life (48), and it is known that beyond 50% of patients following pelvic ring fractures develop chronic pain (49). However, to date, distinct data comparing the pain levels dependent on surgical treatment do not exist. We found a higher pain level in the TIFI group compared to the SPF group (4.2 vs. 1.3; *p* = 0.082) in rest, whereas the pain level under weight-bearing conditions was similar in both groups (4.8 vs. 4.2; *p* = 0.719). These results compare to published pain levels following femur fractures (50).

Group SPF scored better concerning the Merle d'Aubigné (see Table 3), and results are in concordance with published data (20, 41, 43). This might be due to the higher biomechanical stability of the construct. Unfortunately, due to a lack of data in the current literature, we could not compare the Merle d'Aubigné scores of the TIFI group. Regarding the Majeed Pelvic Score, our SPF results compare well to the data of Korrovessis et al. (51), although the ones of the TIFI group do not. This might be because Korrovessis included cases in which an additional iliosacral lag screw was added to the TIFI construct to enhance stability.

As reflected by the SF-36, QoL was reduced compared to the German reference population (32) in both groups and did not show significant differences in the sum scores (PCS/MSC) and the subscores. This compares well to Kerschbaum et al. (27). Similarly, evaluation of SMFA-D did not reveal significant differences between group TIFI and SPF (see Table 4). Jones et al. published SMFA-D data following SPF in complex sacral fractures with lower score levels than our data (47). Again, data following TIFI is so far missing in the literature. However, patient cohorts published and our cohort are heterogeneous regarding the injuries' severities and histories. This makes a direct comparison of those data difficult, and conclusions should be drawn with caution.

Besides the relatively low follow-up of 17.2 months for the clinical evaluation, one of our study's major limitations is its retrospective nature with all its restrictions. Furthermore, we had to use different methods in data acquisition (clinical examination, telephone interview). However, these data represent a single-surgeon series, hence eliminating some bias known from multi-surgeon series. The small number of patients with a clinical follow-up makes it difficult to draw definite conclusions and define recommendations on the treatment of dorsal ring instabilities. However, we saw differences regarding the clinical outcome with favor to SPF. Nevertheless, further studies with long term follow-up are needed to investigate whether the risk of adjunct segment degeneration after SPF might decrease the clinical outcome and lead the surgeon toward other stabilization methods.

Both SPF and TIFI have proven to be valid options to stabilize the posterior pelvic ring. While TIFI tended to have fewer complications, SPF tended toward better clinical results. None of the methods could demonstrate significant superiority over the other. Management of pelvic injuries remains a highly individual challenge adapted to the individual patients' condition. Nevertheless, because of the lower complication rate following TIFI and possible long-term consequences like adjacent segment disease after SPF, TIFI represents a valid option to sufficiently stabilize the posterior pelvic ring in cases the fracture type does not demand spinopelvic fixation.

## Data Availability Statement

The raw data supporting the conclusions of this article will be made available by the authors, without undue reservation.

## Ethics Statement

The studies involving human participants were reviewed and approved by Local Institutional Review Board and the Ethics Commissioner's Vote (No. EA2/036/16). The patients/participants provided their written informed consent to participate in this study.

## Author Contributions

Material preparation, data collection, and analysis were performed by RS, EH, and GR. The first draft of the manuscript was written by RS. All authors commented on previous versions of the manuscript, contributed to the study conception and design, read, and approved the final manuscript.

## Conflict of Interest

The authors declare that the research was conducted in the absence of any commercial or financial relationships that could be construed as a potential conflict of interest.

## Publisher's Note

All claims expressed in this article are solely those of the authors and do not necessarily represent those of their affiliated organizations, or those of the publisher, the editors and the reviewers. Any product that may be evaluated in this article, or claim that may be made by its manufacturer, is not guaranteed or endorsed by the publisher.
